# ‘What’s in a name’, a systematic review of the pterional craniotomy for aneurysm surgery and its many modifications with a proposal for simplified nomenclature

**DOI:** 10.1007/s00701-024-05888-4

**Published:** 2024-01-16

**Authors:** Nicholas G. Candy, Jorn Van Der Veken, Vera Van Velthoven

**Affiliations:** 1 Department of Surgery - Otolaryngology, Head and Neck Surgery, The University of Adelaide, Basil Hetzel Institute for Translational Research, Woodville South, Adelaide, Australia; 2 Department of Neurosurgery, Royal Adelaide Hospital, Adelaide, Australia; 3 Department of Neurosurgery, Aalsters Stedelijk Ziekenhuis, Merestraat 80, 9300 Aalst, Belgium; 4 Department of Neurosurgery, Universitair Ziekenhuis Brussel, Laarbeeklaan 101, 1090 Jette, Belgium

**Keywords:** Pterional craniotomy, Minipterional craniotomy, Lateral supraorbital craniotomy, Aneurysm surgery

## Abstract

**Background:**

The pterional or frontosphenotemporal craniotomy has stood the test of time and continues to be a commonly used method of managing a variety of neurosurgical pathology. Already described in the beginning of the twentieth century and perfected by Yasargil in the 1970s, it has seen many modifications. These modifications have been a normal evolution for most neurosurgeons, tailoring the craniotomy to the patients’ specific anatomy and pathology. Nonetheless, an abundance of variations have appeared in the literature.

**Methods:**

A search strategy was devised according to the 2020 Preferred Reporting Items of Systematic Reviews and Meta-Analyses (PRISMA) statement. To identify articles investigating the variations in the pterional approach, the following search terms were applied: (pterional OR minipterional OR supraorbital) AND (approach OR craniotomy OR technique).

**Results:**

In total, 3552 articles were screened with 74 articles being read in full with 47 articles being included for review. Each article was examined according the name of the technique, temporalis dissection technique, craniotomy technique and approach.

**Conclusion:**

This systematic review gives an overview of the different techniques and modifications to the pterional craniotomy since it was initially described. We advocate for the use of a more standardised nomenclature that focuses on the target zone to simplify the management approach to supratentorial aneurysms.

## Introduction

The pterional or frontosphenotemporal craniotomy remains one of the most used and versatile approaches in vascular (and oncological) neurosurgery, being originally described over 100 years ago [85].

After decades of attempting subfrontal approaches to the sellar region, in 1914, Heuer developed the first frontotemporal craniotomy [61]. However, it was not until the 1950s when the term ‘pterional craniotomy’ was first coined by Hamby. Hamby already emphasised the importance of tailoring the craniotomy to the patient’s specific pathology, a frontolateral approach for anterior communicating artery (ACoA) aneurysms and a frontotemporal craniotomy for middle cerebral artery (MCA) aneurysms [34]. In the 1970s, Yasargil’s perfected and popularised this ‘pterional’ approach with incorporation of the microscope and microsurgical techniques, highlighting the true potential of this approach [85]. This approach has become common practice in modern neurosurgery.

Despite its popularity, there are some well-described disadvantages with this classic technique, particularly temporal muscle wasting with functional and aesthetic consequences including facial asymmetry, discomfort with eyewear, temporomandibular joint dysfunction and mastication pain [7, 35]. A large craniotomy with an extensive exposure of brain cortex also increases the risk of iatrogenic injury; hence, customising the pterional craniotomy to a patient’s specific pathology has been a natural evolution for many surgeons. Furthermore, the general principle of the relation between the pterional craniotomy and the Sylvian fissure cannot be overemphasised [5, 86].

Over the last decades, many variants have been described. This review examines the different described techniques, discusses their adaptations and advocates for adoption of standardised nomenclature.

## Methods

### Literature search

A search strategy was devised according to the 2020 Preferred Reporting Items of Systematic Reviews and Meta-Analyses (PRISMA) statement [63]. An electronic search of the databases Medline, Scopus, Embase, Web of Science and Cochrane library databases was performed from 1st January 1956 to 17th of July 2023. Articles were limited to the English language. To identify articles investigating the variations in the pterional approach, the following search terms were applied: (pterional OR minipterional OR supraorbital) AND (approach OR craniotomy OR technique) with prior checking in the MeSH database to include synonyms.

The database search was further supplemented by a search of the reference lists of included studies as well as checking the related article function provided by each database. Titles and abstracts were screened to identify potentially relevant studies. All potentially relevant articles, or articles where it was unclear based on the abstract, were assessed by reviews of the full-text articles.

Articles were deemed eligible if they: (1) specifically described the surgical technique in detail; (2) state the technique is a unique modification or novel technique and use unique nomenclature that differentiates it from previous iterations; (3) published in a peer-reviewed journal; (4) the approach is specifically designed for vascular pathology managed microscopically or with endoscopic assistance; (5) the technique was indicated for management of aneurysms of the supraclinoid internal carotid artery, proximal middle cerebral artery and proximal anterior cerebral artery/anterior communicating artery complex.

Article were excluded when: (1) the primary goal of the article was not presenting a surgical technique; (2) they present a novel subdural and subarachnoid corridor but use a previously described craniotomy; (3) only demonstrate technique in cadaver specimens; (4) article did not undergo peer review, such as a letter to the editor; (5) the approach has only been used to treat neoplastic pathology; (6) article only presents management of soft tissue dissection without a novel craniotomy; (7) article describes a pure transcranial endoscopic approach; (8) the focus was posterior circulation, distal anterior cerebral artery or distal middle cerebral artery aneurysms.

### Data extraction

All data was reviewed independently by two authors (NC and JV) and discrepancies cross checked in a consensus meeting.

The following data was obtained from the included studies: title and year of publication, name of the approach, type of skin incision, described craniotomy location, true anatomical location of the craniotomy, dural incision, approach, target zone exposure or indication for treatment of what type of aneurysms.

### Quality assessment

We used a modified quality assessment tool incorporating the Cochrane Collaboration tool to assess the methodological quality of the included articles [37]. The quality assessment tool (Table 1) assessed the following: demographic details, pre-operative variables, post-operative variables, complications and learning curve. The same two authors (NC and JV) then evaluated the risk of bias in the individual articles using a modified version of the Cochrane Collaboration method (Table 2). Discrepancies were resolved after discussion and consensus amongst all authors.

**Table 1:** Quality assessment tool

*Quality category*	*Questions*	*Response*		
		Yes	No	Unclear
Literature review	Is there a relevant review of the background in the field?			
	Do the authors clearly outline why this novel approach is needed?			
	Do the authors clearly outline what makes this approach different to what has already been published?			
Surgical technique	Is the relevant surgical anatomy reviewed?			
	Is the description outlined in a logical stepwise fashion?			
	Do the authors include representative diagrams or photos?			
	Is the anatomical target zone achieved by this approach defined clearly?			
Case series	Do the authors include a case series that utilised this novel approach?			
	Are the cases complications clearly discussed			
Evaluation	Do the authors review the advantages and disadvantages of this approach?			
	Do the authors define the indications and contraindications for this approach?

**Table 2:** Grading of quality assessment

*Quality category*	Poor	Moderate	Good
Literature review	$\le 1\mathrm{ criteria}$	$\le 2\mathrm{ criteria}$	$3\mathrm{ criteria}$
Surgical technique	$\le 2\mathrm{ criteria}$	$\le 3\mathrm{ criteria}$	$4\mathrm{ criteria}$
Case series	$\le 1\mathrm{ criteria}$		$2\mathrm{ criteria}$
Evaluation	$\le 1\mathrm{ criteria}$		$2\mathrm{ criteria}$

## Results

### Study selection

**Fig. 1:**
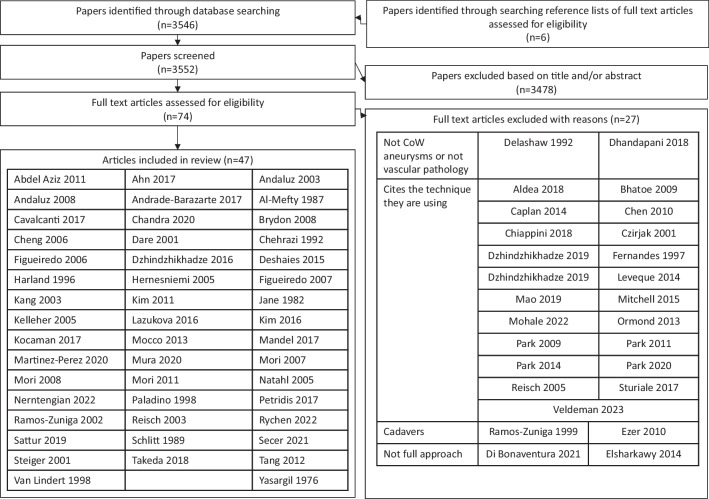
PRISMA flowchart demonstrating the process of study selection

From the literature search, 3546 articles were identified through searching Medline, Embase, Scopus, Web of Science and Cochrane library databases. Six additional articles were also identified by screening reference lists of included articles and searching the recommended article section of applicable databases. In total, 3552 articles were screened with 74 articles being read in full and 27 of these articles being excluded. The common reason for exclusion involved 21 articles [4, 9, 11, 16, 18, 19, 26, 27, 30, 46, 49, 52, 54, 62, 65–68, 73, 80, 84] and is because the authors cited another article when describing the surgical technique. The other reasons for exclusion included as follows: one article [23] designing an approach to treat distal anterior cerebral artery aneurysms, one article [21] designed an approach that was not for cerebrovascular pathology, two articles [29, 71] only described their technique being used in cadavers and two articles [24, 28] only described a new subdural corridor without a novel craniotomy. Figure 1 contains the flowchart of study selection. The included 47 articles [1–3, 6–8, 10, 12, 14, 15, 17, 20, 22, 25, 31, 32, 35, 36, 38–43, 45, 48, 51, 53, 55–60, 64, 70, 72, 74–79, 81–83, 85] were reviewed in detail.

### Study characteristics

#### 1976

Yasargil et al. [85] describe the *fronto-spheno-temporal craniotomy* which involves an incision starting 1 cm superior to the anterior aspect of the auricle and extends to the temporal crest in a direction perpendicular to the zygoma ending at the widows peak. Four burrholes are described, being located just superior to the frontal zygomatic suture under the linea temporalis, second burrhole in the frontal bone 3–4 cm above it, third burrhole in the parietal bone along the linea temporalis and fourth burrhole in the squamous temporal bone behind the spheno-temporal line. They term this the ‘*pterional’ craniotomy*’ and it gives a subfrontal and transsylvian approach to aneurysms of the anterior circulation.

#### 1982

Jane et al. [38] describe the *supraorbital approach* which involves a bicoronal incision with a fronto-spheno-zygomatic craniotomy requiring a burrhole at the midline level of the orbital ridge and a second burrhole just behind the zygomatic process. This grants a subfrontal approach to orbital tumours, ACoA aneurysms, pituitary tumours, craniopharyngiomas, parasellar and olfactory groove meningiomas.

#### 1987

Al-Mefty [3] described the *supraorbital-pterional approach* to manage large central skull base lesions, including giant basilar aneurysms. The approach involves a bicoronal incision with a single piece craniotomy involving fronto-spheno-zygomatico-temporal bone, including the orbital rim.

#### 1989

Schlitt et al. [77] describe the *osteoplastic pterional craniotomy* which is a fronto-spheno-temporal osteoplastic craniotomy that starts with an incision at the zygomatic process 2 cm anterior to the external auditory canal and curves frontally towards the lateral third of the eyebrow. The first burrhole is posterior and inferior to the anterior point of the superior temporal line, second burrhole posterior temporal above the zygomatic root. A rongeur is used craniectomies the remaining bone and fracturing across the gap. They do not define the approach, but describe the approach as granting access to internal carotid artery (ICA), ACoA and MCA aneurysms.

#### 1992

Chehrazi et al. [15] described the *temporal transsylvian approach*. This started with a skin incision at the zygomatic arch 1 cm anterior to the auricle and extending superiorly and anteriorly to the superior extent of the ‘keyhole’. A single burrhole is placed over the exposed sphenoid wing of the sphenoid with a fronto-spheno-temporal craniotomy being raised. This grants a transsylvian corridor and allows treatment of all aneurysms of the anterior circulation.

#### 1996

Hardland et al. [35] described the *modified pterional***,** which involved a 5–6-cm curvilinear incision immediately behind the hairline from the superior temporal line to the midpoint of the zygomatic arch. A single burrhole is placed over the pterion and this is extended as a craniectomy using rongeurs, removing fronto-spheno-temporal bone. This grants a transsylvian corridor and allows treatment of MCA, ACA, posterior communicating artery (PCoA), ophthalmic and terminal carotid aneurysms.

#### 1998

Van Lindert et al. [83] described the *supraorbital keyhole approach*. The skin incision starts lateral to the supraorbital nerve and finishes at the lateral edge of the eyebrow in front of the zygomatic process. A single burrhole is placed in the temporal fossa just behind the superior temporal line. The subsequent craniotomy can have three variants depending on the target area to be reached and includes as follows: frontal, fronto-sphenoidal and fronto-spheno-temporal. Through a subfrontal corridor, all aneurysms of the anterior circulation can be reached.

Paladino et al. [64] also described the *eyebrow keyhole approach* with a skin incision that is poisitoned in the lateral two-thirds of the eyebrow. A single burrhole is made 1 cm above the supraorbital rim and 1 cm lateral to the supraorbital nerve to raise a frontal craniotomy. This grants a subfrontal corridor and allows treatment of ACoA and anterior choroidal aneurysms.

#### 2001

Steiger et al. [79] describe the *transorbital keyhole approach* with an incision that starts 1 cm in front of the tragus and slightly above the zygomatic arch, extending frontally towards the midline. The first burrhole is placed over the keyhole of the pterional craniotomy, and the second is placed above the upper orbital rim at the medial aspect of the planned craniotomy. This raises a fronto-spheno-zygomatic craniotomy granting a subfrontal corridor to treat ACoA aneurysms.

Dare et al. [20] also described the *eyebrow incision-minisupraorbital craniotomy with orbital osteotomy.*

#### 2002

Ramos-Zuniga et al. [72] described the *trans-supraorbital approach* involving a 3-cm incision made through the eyebrow between the pupil median line and the external rim of the zygomatico-orbital joint. They describe an en bloc craniotomy within the supraorbital foramen, zygomatico-orbital joint, orbital arch with a 1-cm extension into the depth of the orbital roof. This raises a frontal craniotomy which grants a subfrontal corridor for treatment of aneurysms on the ICA, ACoA and MCA.

#### 2003

Andaluz et al. [7] describe the *one piece orbitopterional approach*. Involving a hair sparing incision starting 1 cm below the zygoma and follows the hairline approximately 3 cm beyond the midline. The first burrhole is at the frontosphenoidal suture about 1 cm behind the frontozygomatic junction. Second burrhole is superior to the root of the zygoma, and these are connected to raise an orbitopterional craniotomy involving fronto-spheno-zygomatico-temporal bones. This grants a subfrontal corridor and allows access to the ipsilateral optic nerve, optico-carotid cistern, ACoA and potential suprasellar and infrachiasmatic tumours.

Kang et al. [39] described a *pterional craniotomy with keyhole* for treatment of supratentorial aneurysms. This variant involved a single burrhole in the posterior temporal fossa.

Reisch et al. [74] describe the *supraorbital keyhole craniotomy* (Table 3).

**Table 3:** Study characteristics of the included articles

	Year of publication	Name of approach	Skin incision	Temporalis dissection	Described craniotomy location	Anatomical craniotomy location	Approach	Target zone exposure or indications for aneurysms
Yasargil	1976	Pterional craniotomy	1 cm superior to the anterior aspect of the auricle and extends to the temporal crest in a direction perpendicular to the zygoma	Interfascial dissection	First burrhole just superior to the frontal zygomatic suture under the linea temporalis, second burrhole in the frontal bone 3–4 cm above it, third burrhole in the parietal bone along the linea temporalis, fourth burrhole squamous temporal bone behind the spheno-temporal line	Fronto-spheno-temporal	Subfrontal and transsylvian	Anterior circulation aneurysms
Jane et al	1982	Supraorbital approach	Bicoronal incision	Not defined	First burrhole midline at the level of the orbital ridge. Second burrhole just behind the zygomatic process	Fronto-spheno-zygomatic	Subfrontal	Approach orbital tumours, ACoA aneurysms, pituitary tumours, craniopharyngiomas, parasellar and olfactory groove meningiomas
Al-Mefty	1987	Supraorbital-pterional approach to skull base lesions	Bicoronal incision	Not defined	First burrhole in the frontal bone above the nasion, second burrhole MacCarty keyole, third burrhole posterior temporal near temporal fossa floor. Single bone flap involving orbital rim	Fronto-spheno-zygomatico-temporal	Subfrontal, transsylvian, subtemporal	Large central skull base lesions and giant basilar artery aneurysms
Schlitt et al	1989	Osteoplastic pterional craniotomy	Starting at the zygomatic process 2 cm anterior the external auditory canal, curved frontally towards the lateral third of the eyebrow	Osteoplastic	First burrhole posterior and inferior to the anterior point of the superior temporal line. Second burrhole posterior temporal above the zygomatic root. Rongeur to craniectomise bone towards the burrholes and then fracturing across the gap. Raising an osteoplastic flap	Fronto-spheno-temporal	Undefined	Access ICA, ACA and MCA aneurysms
Chehrazi et al	1992	Temporal transsylvian approach	Zygomatic arch 1 cm anterior to the auricle extending superiorly and anteriorly to the superior extent of the ‘keyhole’	Muscle splitting and subperiosteal dissection	First burrhole over the exposed wing of the sphenoid. Dura over the frontal and temporal aspects of the sphenoid wing is exposed with the craniotomy being centred over the Sylvian. 3 × 4 cm size	Fronto-spheno-temporal	Transsylvian	All aneurysms of the anterior circulation
Harland et al	1996	Modified pterional	5–6 curvilinear incision immediately behind the hair line from the superior temporal line to the midpoint of the zygomatic arch	Muscle splitting and subperiosteal dissection	First burrhole over pterion. Extended as a craniectomy with rongeur	Fronto-spheno-temporal	Transsylvian	MCA, ACA, PCoA, ophthalmic and terminal carotid aneurysms treated
Van Lindert et al	1998	Supraorbital keyhole approach	Starting laterally from the supraorbital nerve and finishing at the lateral edge of the eyebrow in front of the zygomatic process	Muscle splitting and subperiosteal dissection	First burrhole temporal fossa just behind the superior temporal line. Bone flap raised with three potential variants	Frontal, fronto-sphenoidal, fronto-spheno-temporal	Subfrontal	All supratentorial anterior circulation aneurysms
Paladino et al	1998	Eyebrow keyhole approach	Linear skin incision positioned in the lateral 2/3 of the eyebrow	Muscle splitting and subperiosteal dissection	First burrhole 1 cm above the supraorbital rim and 1 cm lateral to the supraorbital nerve. Small 15 × 25 mm osteoclastic craniotomy	Frontal	Subfrontal	ACoA, anterior choroidal
Steiger et al	2001	Transorbital keyhole approach	Started 1 cm in front of the tragus and slightly above the zygomatic arch. Extended frontally towards the midline	Muscle splitting and subperiosteal dissection	First burrhole over the keyhole of the pterional craniotomy. Second burrhole placed above the upper orbital rim at the medial aspect of the planned craniotomy	Fronto-spheno-zygomatic	Subfrontal	Interhemispheric fissure approach ACoA
Dare et al	2001	Eyebrow incision-minisupraorbital craniotomy with orbital osteotomy	Placed in the superior edge of the eyebrow, starting from the midpupillary line and extending laterally to just behind the frontal process of the zygomatic bone	Muscle splitting and subperiosteal dissection	First burrhole a few mm above the frontosphenoid suture. A single piece bone flap raised measuring 2.5 cm × 3.5 cm	Fronto-spheno-zygomatic	Subfrontal	MCA, anterior choroidal, PCoA, ACoA, ICA bifurcation aneurysms
Ramos-Zuniga et al	2002	Trans-supraorbital approach	3-cm incision made through the eyebrow between the pupil median line and the external rim of the zygomatic-orbital joint	Muscle splitting and subperiosteal dissection	En bloc craniotomy within supraorbital foramen, zygomatico-orbital joint, orbital arch with 1-cm extension into the depth of the orbital roof	Frontal	Subfrontal	Aneurysms on the ICA, ACoA and MCA
Reisch et al	2003	Supraorbital keyhole craniotomy	Incision within the eyebrow lateral to the supraorbital nerve extending a few mm beyond the lateral edge of the eyebrow	Subperiosteal dissection	First burrhole. Frontobasal posterior to the temporal line accessing anterior fossa. Frontal craniotomy 20–25-mm wide and 15–20-mm high	Frontal	Subfrontal	Undefined
Andaluz et al	2003	One-piece orbitopterional approach	Hair-sparing incision. 1 cm below the zygoma and follows the hairline ~ 3 cm beyond midline on the contralateral side	Subfascial dissection	First burrhole frontosphenoidal suture, ~ 1 cm behind frontozygomatic junction. Second burrhole superior to the root of zygoma. One piece orbitopterional craniotomy	Fronto-spheno-zygomatico-temporal	Subfrontal	Ipsilateral optic nerve, optico-carotid cistern. Approach ACoA aneurysms and suprasellar or infrachiasmatic tumours
Kang et al	2003	Pterional craniotomy without keyhole	Same as standard pterional	As per standard pterional	First burrhole temporal fossa. Single bone flap	Fronto-spheno-temporal	Subfrontal and transsylvian	Supratentorial aneurysms
Nathal et al	2005	Sphenoid ridge approach	4–5-cm incision at the level of the hairline centred at the estimated location of the sphenoid ridge	Separate muscle flap in plane with skin incision	First burrhole most caudal aspect of the surgical exposure centred over the bony depression representing the sphenoid ridge. Oval-shaped craniotomy over the sphenoid wing	Fronto-spheno-temporal	Transsylvian	Treat aneurysms of paraclinoid ICA, PCoA, anterior choroidal artery, ICA birfurcation, MCA and ACoA
Hernesniemi et al	2005	Lateral supraorbital approach	Frontotemporal incision behind the hairline. Does not go as low as in front of the ear	Myocutaneous flap	First burrhole. Posteriorly just below the insertion of the temporalis muscle. Bone flap 3 × 3 cm to 4 × 4 cm	Fronto-sphenoidal	Subfrontal and transsylvian	Whole anterior part of the anterior circle of willis, sellar, suprasellar region and anterior part of the basilar artery
Kelleher et al	2005	Cranio-orbital approach	Skin incision begins below zygoma 1 cm anterior to the auricle and extended superiorly across the midline posterior to the hairline	Subfascial dissection	First burrhole at keyhole, second burrhole at low postero-temporal, third burrhole superior temporal just above the superior temporal line and fourth burrhole low frontal and lateral to supraorbital notch. Raise a single piece cranio-orbital flap	Fronto-spheno-temporo-zygomatic	Subfrontal and transsylvian	MCA, carotid bifurcation, ACoA, ophthalmic, basilar aneurysms
Figueiredo et al	2006	Supraorbital minimodified orbitozygomatic craniotomy	Arcuate shape starting at the base of the zygomatic arch 1 cm anterior to the tragus extended to the contralateral midpupillary line	Muscle splitting and subperiosteal dissection	Burrhole at MacCarty keyhole. Boneflap lateral to the supraorbital notch involving orbital bar and zygomatic process of the frontal bone	Fronto-spheno-zygomatic	Transslyvian and subfrontal	Ipsilateral MCA bifurcation, ipsilateral ICA birfurcation, basilar artery bifurcation, contralateral ICA bifurcation, ACoA, contralateral MCA
Cheng et al	2006	Pterion minicraniotomy	Oblique incision 3–5 cm in length 1 cm anterior to the STA at the level of the zygomatic arch anteriorly curved below the temporal line towards the forehead	Myocutaneous flap	First burrhole temporal bone near the posterior margin of the zygomatic arch. Craniotomy width 2–3.5 cm and height 1.5–2 cm created, limited by the sphenoid ridge anteriorly, suprameatal crest posteriorly, zygomatic arch inferiorly and squamous suture superiorly	Temporo-sphenoidal (with option for frontal extension)	Transsylvian	Access to ipsilateral ICA, medial wall of contralateral ICA, ACoA, bilateral ophthalmic arteries, M1, M2 and M3 segments of ipsilateral MCA, contralateral PCoA, anterior choroidal, P1 PCA, suprasellar and tentorial areas, all anterior cranial base structures
Figueiredo et al	2007	Minipterional craniotomy	Arcuate scalp incision, starting 1 cm above the base of the zygomatic arch at the anterior border of the hairline. Extending superiorly and curving towards the ipsilateral midpupillary line	Interfascial dissection	First burrhole superior to the frontozygomatic suture under linea temporalis. At the stephanion, it curves inferiorly to include the pterion. Then anteriorly inferiorly along the sphenoid bone to connect back to the burrhole	Fronto-spheno-temporal	Transsylvian and subfrontal	Not statistically different to pterional
Mori et al	2007	Pterional keyhole craniotomy through an outer canthal incision	Curved incision 30–35-mm long along the lateral margin of the orbit from the outer aspect of the eyebrow slightly lateral to the anterior temporal line to a point 10 mm superior to the zygomatic arch	Muscle splitting and subperiosteal dissection	First burrhole at the pterion. Circular craniotomy raised 20–25-mm diameter	Fronto-spheno-temporal	Transsylvian	MCA aneurysms
Mori et al	2008	Lateral supraorbital approach via a periorbital skin incision	Curved incision 40–50 mm long starting at the lateral part of the eyebrow and along the lateral margin of the orbit to a point 15 mm superior to the zygomatic arch	Muscle splitting and subperiosteal dissection	First burrhole lateral to MacCarty keyhole, just above the frontosphenoid suture. Minicraniotomy 35 × 25 mm	Fronto-sphenoidal	Subfrontal	ACoA and ACA aneurysms
Andaluz et al	2008	Transeyelid, supratarsal, transorbital roof minicraniotomy	2.5–3.5-cm upper eyelid incision along the eyelid crease. 10 mm superior to the upper lid margin and 6 mm above the lateral canthus at its lateral extent	Muscle splitting and subperiosteal dissection	First burrhole frontosphenoidal junction, MacCarty keyhole. One piece transorbital roof supraorbital minicraniotomy	Fronto-spheno-zygomatic	Subfrontal	Ipsilateral optic nerve, opticocardotid cistern
Brydon et al	2008	Supra-orbital microcraniotomy	< 1 cm above the eyebrow from the supra-orbital notch to the anterior border of the infratemporal fossa (?temporal)	N/A	8 mm above the supraorbital rim a rectangular craniotomy is made without burrholes	Frontal	Subfrontal	Not defined
Kim et al	2011	Osteomyoplastic monoblock pterional craniotomy	As per standard pterional	Osteoplastic	Keyhole burrhole 2 cm posterosuperiorly to the pterion on the Sylvian fissure. Superior cut from burrhole along frontal bone until limited by temporalis. Inferior cut from burrhole towards sphenoid ridge. Sphenoid ridge osteotomy	Fronto-spheno-parieto-temporal	Subfrontal and transsylvian	Not defined
Abdel Aziz et al	2011	Transpalpebral approach	Superior eyelid through upper eyelid crease up to 2.5 cm lateral to the lateral canthus	Muscle splitting and subperiosteal dissection	Burrhole greater wing of sphenoid and junction between lateral wall and roof of orbit. One piece fronto-orbital craniotomy	Fronto-spheno-zygomatic	Subfrontal	Optic cistern, carotid cistern, proximal Sylvian fissure, ipsilateral and contralateral oculomotor nerve
Mori et al	2011	Individualised pterional keyhole based on 3D virtual osteotomy	W-shaped incision 4-cm length made from the superior temporal line to 1 cm above the zygomatic arch	Muscle splitting and subperiosteal dissection	First burrhole on pterion, 25 mm craniectomy on pre-planned location	Fronto-sphenoidal	Trassylvian	MCA aneurysms
Mocco et al	2013	Minimally invasive pterional keyhole approach	Curvilinear hockey stick or question mark incision starting 5 mm anterior to the tragus and 2 mm above the zygomatic root. Extending up to the temporal line behind the hairline	Interfascial dissection	First burrhole most inferior portion of the exposed bone superior to the frontozygomatic suture. Elliptical craniotomy 2.5 × 4 cm	Fronto-spheno-temporal	Transsylvian	Aneurysms of the anterior circulation including as follows: ACA, ACoA, ophthalmic, internal carotid bifurcation, MCA bifurcation
Tang et al	2013	Modified supraorbital keyhole approach	Made in a skin crease or the eyebrow, avoiding supraorbital nerve	Muscle splitting and subperiosteal dissection	First burrhole placed below the superior temporal line and posterior to the keyhole. Fifteen to 20 mm by 20–25 mm craniotomy is raised	Fronto-sphenoidal	Subfrontal	ACA, ICA, MCA aneurysms
Deshaies et al	2015	Minimally invasive thumb sized pterional craniotomy technique	Midline of the scalp extending behind the hairline with a gentle curve posteriorly and inferiorly towards the tragus ending about 3 mm anterior to the tragus at the superior edge of the zygoma	Myocutaneous flap	First burrhole temporal posteriorly. Three to 4 cm craniotomy kidney-shaped	Fronto-spheno-temporal	Transsylvian and subfrontal	Supraclinoidal aneurysms
Kim et al	2016	Modified supraorbital keyhole approach	Conventional pterional skin incision	Interfascial dissection	First burrhole. 0.5 cm down from the frontozygomatic suture. 3 × 3 cm frontotemporal craniotomy made	Fronto-spheno-temporal	Subfrontal	Not defined
Lazukova et al	2016	Modified lateral supraorbital approach	As per the lateral supraorbital approach	As per LSO	Same as lateral supraorbital	Fronto-sphenoidal	Subfrontal	Anterior cranial fossa pathology and anterior circulation aneurysms
Dzhindzhikhadze et al	2016	Mini-orbitozygomatic craniotomy	Eyebrow incision from pupillary line laterally within the eyebrow, sometimes extending beyond	Subperiosteal dissection	Burrhole posterior to the temporal line above the base of the anterior cranial fossa. Single bone flap involving frontal bone, roof of the orbit and zygomatic bone	Fronto-spheno-zygomatic	Transsylvian and subfrontal	Access to Sylvian fissure, optico-carotid cistern, chiasmal cistern, Liliequist membrane, terminal plate of the third ventricle, suprasellar and parasellar cisterns
Mandel et al	2017	Modified transpalpebral, supratarsal, transorbital roof minicraniotomy	Skin in the upper lid crease from the mid pupillary line until the lateral canthal angle	N/A	First burrhole spheno-orbital keyhole. One piece mini fronto-orbital craniotomy	Fronto-spheno-zygomatic	Subfrontal	Used exclusively for MCA aneurysms
Ahn et al	2017	Superficial temporal artery sparing mini pterional approach	Curvilinear starting above STA bifurcation towards the hairline	Interfascial dissection	First burrhole at keyhole, second burrhole below temporal squamous suture. Craniotomy 3 × 4 cm	Fronto-spheno-temporal	Transsylvian	As wide as the classic pterional
Andrade-Barazarte et al	2017	Extended lateral supraorbital craniotomy and extradural anterior clinoidectomy	Short curvilinear frontotemporal. Staying above the front of the ear	As per standard LSO	First burrhole posteriorly just below the insertion line of the temporal muscle. Craniotomy size 3 × 3 to 4 × 4 cm. Extradural dissection and drilling with subsequent anterior clinoidectomy	Fronto-sphenoidal	Subfrontal	Access internal carotid artery aneurysms, anterior skull base lesions including those in cavernous sinus and temporomesial region
Petridis et al	2017	Modified mini pterional subfrontal supratentorial approach	5-cm incision curvilinear frontotemporal starting above the zygomatic root	Incised along STL and subperiosteal dissection	Two burrholes and a 2.5 cm craniotomy is raised over the pterion	Fronto-spheno-temporal	Subfrontal	MCA bifurcation aneurysms
Cavalcanti et al	2017	Minisphenoidal approach	Behind hairline, two fingerbreadths behind frontozygomatic suture, 1 cm in front of the top of the pinna, arching medially 6–7 cm	Myocutaneous flap	First burrhole posteriorly over the depression corresponding internally to the sphenoid ridge with a small bean shaped craniotomy being raised. V-shaped trough drilled over the sphenoid ridge. Craniotomy 3 cm horizontally and 3–4 cm vertically	Fronto-spheno-temporal	Transsylvian	Initially simple MCA aneurysms, expanded to include most aneurysms accessed through pterional
Takeda et al	2018	Distal transsylvian keyhole approach	7-cm incision beginning at the zygoma curving frontally to the lateral canthus	Subfascial dissection	First burrhole MacCarty keyhole. Second burrhole temporal bone. Circular craniotomy over the sphenoid ridge	Fronto-spheno-temporal	Transsylvian	MCA bifurcation, laterally and posteriorly projected PCoA aneurysms, anterior choroidal aneurysms
Kocaman et al	2018	Modified lateral supraorbital approach	Begins just above the zygomatic arch, passing medially behind the hairline just beyond midline	Muscle splitting and subperiosteal dissection	First burrhole. MacCarty keyhole. Frontal craniotomy 3–4 × 3–4 cm in size	Frontal	Subfrontal	Optic cistern, Sylvian cistern, opposite Sylvian fissure
Chandra et al	2020	Fronto-orbital variant of the supra-orbital keyhole craniotomy (f-SOKHA)	Lower part of the eyebrow starting lateral to the supraorbital foramen extending to just behind the frontal process of the zygomatic arch	Muscle splitting and subperiosteal dissection	Medial inferior edge of the craniotomy went around the level of the frontal base and the lateral edge to the sphenoid wing, creating a size of 3 × 2.5 cm	Fronto-sphenoidal	Subfrontal	ACoA, PComA, MCA, supra-clinoid, paraclinoid
Martinez-Perez et al	2020	Extradural minipterional approach	Incised 1 cm behind the hairline between the mid-pupillary line and the zygoma	Interfascial dissection	Minipterional craniotomy, as previously described. Partial osteotomy of the orbit. Extradural anterior clinoidectomy	Fronto-spheno-temporal	Transsylvian	Non-ruptured complex aneurysms in the anterior circulation
Sattur et al	2020	Extended lateral orbital approach	Middle of the eyebrow medial to the superior temporal line, curving laterally and inferiorly with the lateral orbital rim 1.5 cm lateral to the inner rim or later canthus. Then zigzag over the anterior origin of the zygomatic arch	Muscle splitting and subperiosteal dissection	First burrhole over keyhole and second burrhole at inferior limit of bony exposure. Connected to complete craniotomy	Fronto-spheno-temporal	Transsylvian and subfrontal	Ipsilateral structures were A1, proximal A2, recurrent artery of Heubner’s, supraclinoid and terminal ICA, optic nerve, falciform ligament, oculomotor nerve, PCoA, and anterior choroidal artery, superior cerebellar artery, P1 segment of the PCA, MCA bifurcation. Midline structures were ACoA, superior optic chiasm, basilar apex. Contralateral structures were A1, carotid terminus, PCoA, M1 and MCA bifiurcation, and carotid terminus
Mura et al	2020	Extradural minipterional approach	2 cm above the superior edge of the zygomatic arch. Follows the anterior insertion of the hair to the hemi-pupil line	Interfascial dissection	Bone groove performed 0.5 cm below the superior temporal line behind the zygomatic process of the frontal bone. Craniotomy upper limit in the superior temporal line. Optional extradural anterior clinoidectomy	Fronto-spheno-temporal	Transsylvian	Aneurysms of the anterior circulation. Tumours of the parasellar, meckel’s cave and interpeduncular fossa
Nerntengian et al	2022	Mini-spheno-supraorbital craniotomy	Typical curvilinear frontotemporal incision beginning slightly anterior to tragus and reaching the widows peak at the midline	Subfascial dissection	First burrhole at the keyhole as described by Yasargil. Ellipsoid shaped bone flap with its long axis being in coronal and its short axis in AP plane was elevated in the spheno-supraorbital region	Fronto-sphenoid	Subfrontal and transsylvian	Treatment of anterior circulation aneurysms
Rychen et al	2022	Sylvian keyhole approach	4.5-cm curvilinear incision behind the temporal hairline	T-shaped incision with subperiosteal dissection	First burrhole inferior aspect of exposed bone. 2.5 × 2.5 cm craniotomy. Typically centred at the anterior squamosal point	Fronto-temporal	Transsylvian	MCA aneurysms
Secer et al	2022	Modified osteoplastic pterional craniotomy	Semilunar incision started from the front of the tragus curved forwards frontally	Osteoplastic	First burrhole MacCarty keyhole. Second burrhole posterior temporal. Circular craniotomy connecting the two burrholes. Fracture across the bone bridge	Fronto-spheno-temporal	Subfrontal and transsylvian	Same as pterional craniotomy

Absolute number reported if available, and then percentage of cohort

*STA* superficial temporal artery, *ICA* internal carotid artery, *MCA* middle cerebral artery, *PCoA* posterior communicating artery, *ACoA* anterior communicating artery, *ACA* anterior cerebral artery, *PCA* posterior cerebral artery, *N/A* not applicable

#### 2005

Hernesniemi et al. [36] describe the *lateral supraorbital (LSO)* approach involving a frontotemporal incision behind the hairline and does not go as low as the conventional pterional incision. A single burrhole is placed posteriorly just below the insertion of the temporalis muscle. This allows elevation of a fronto-sphenoidal craniotomy granting a subfrontal and transsylvian corridor allowing access to the whole anterior part of the anterior circle of Willis, sellar, suprasellar region and anterior part of the basilar artery.

Nathal et al. [59] described the *sphenoid ridge approach*.

Kelleher et al. [40] described the *cranio-orbital approach* (Table 3).

#### 2006

Figueiredo et al. [31] described the *supraorbital minimodified orbitozygomatic craniotomy*. Involving an arcuate incision starting at the base of the zygomatic arch 1 cm anterior to the tragus extending to the contralateral midpupillary line. A single burrhole at MacCarty keyhole and then raising a craniotomy involving the fronto-spheno-zygomatic bone. Grants a transsylvian and subfrontal corridor allowing access to the ipsilateral MCA bifurcation, ipsilateral ICA bifurcation, basilar artery bifurcation, contralateral ICA bifurcation, ACoA and contralateral MCA.

Cheng et al. [17] also described the *pterion minicraniotomy*.

#### 2007

Figueiredo et al. [32] described the *mini-pterional craniotomy*. This approach involved an arcuate incision starting 1 cm above the base of the zygomatic arch at the anterior border of the hairline and extending superiorly and curving towards the ipsilateral mid-pupillary line. A single burrhole is made superior to the frontozygomatic suture under the linea temporalis. A circular craniotomy is commenced by carrying the craniotome posteriorly and at the stephanion curving inferiorly to include the pterion then curving anteriorly back to connect with the burrhole. This raises a fronto-spheno-temporal craniotomy and allows a transsylvian and subfrontal corridor. The authors found no statistically significant difference between this exposure and that of a conventional pterional.

Mori et al. [56] also described the *pterional keyhole craniotomy* through an outer canthal incision (Table 3).

#### 2008

Andaluz et al. [6] described the *transeyelid*, *supratarsal*, *transorbital roof minicraniotomy*. Involving an incision of 2.5 to 3.5 cm in the upper eyelid along the eyelid crease, 10 mm superior to the upper lid margin and 6 mm above the lateral canthus at its lateral extent. A single burrhole at the MacCarty keyhole and then raising a fronto-spheno-zygomatic craniotomy. This allows a subfrontal corridor to access the ipsilateral optic nerve and optico-carotid cistern.

Mori et al. [57] described the *lateral supraorbital approach *via* a periorbital skin incision*.

Brydon et al. [10] described the *supra-orbital minicraniotomy* (Table 3).

#### 2011

Kim et al. [41] described the *osteomyoplastic monoblock pterional craniotomy* which involved a fronto-spheno-parieto-temporal craniotomy with temporalis muscle still attached following a keyhole burrhole, superior osteotomy along the frontal bone limited by temporalis and an inferior osteotomy along the squamosal temporal bone.

Abdel Aziz et al. [1] described the *transpalpebral approach* which involved an incision through the superior eyelid crease up to 2.5 cm from the lateral canthus and 1 piece fronto-orbital craniotomy.

Mori et al. [55] described the *individualised pterional keyhole based on 3D virtual osteotomy*. This involved a W shaped made in front of the hairline with a burrhole over the pterion and a further 25 mm craniectomy based on pre-planned location. This created a transsylvian corridor to treat MCA aneurysms.

#### 2013

Mocco et al. [53] described the *minimally invasive pterional keyhole* approach starting with a hockeystick incision anterior to the tragus and above the posterior root of zygoma extending up towards the temporal line staying behind the hairline. An elliptical craniotomy is raised that grants a transsylvian corridor.

Tang et al. [82] described the *modified supraorbital keyhole* approach which is started with a skin incision made into the skin crease or the eyebrow and creating a fronto-sphenoidal craniotomy.

#### 2015

Deshaies et al. [22] described the *minimally invasive thumb sized pterional* craniotomy technique which involves an incision starting at the midline behind the hairline extending towards the tragus in a gentle curve ending 3 mm anterior to the tragus. A 3–4 cm fronto-spheno-temporal craniotomy is raised.

#### 2016

Kim et al. [42] described the *modified supraorbital keyhole approach* which is started with a conventional pterional skin incision and involves a fronto-spheno-temporal craniotomy.

Lazukova et al. [45] described the *modified lateral supraorbital approach* which is the same as the lateral supraorbital approach except for the application of an orbitozygomatic stitch.

Dzhindzhikha et al. [25] described the *mini-orbitozygomatic craniotomy* started with an eyebrow incision from the pupillary within the eyebrow, sometime extending beyond. Raising a single bone flap involving frontal bone, roof of orbit and zygomatic bone.

#### 2017

Mandel et al. [48] described the *modified transpalpebral*, *supratarsal*, *transorbital roof minicraniotomy* involving a skin incision in the upper lid crease from mid pupillary line to the lateral canthal angle. A mini fronto-orbital craniotomy is raised that involves frontal, sphenoid and zygomatic bones.

Ahn et al. [2] described the *superficial temporal artery sparing mini pterional approach* which involves a curvilinear incision starting above the STA bifurcation.

Andrade-Barazarte et al.[8] described the *extended lateral supraorbital craniotomy and extradural anterior clinoidectomy.*

Petridis et al. [70] described the *modified mini pterional subfrontal supratentorial approach*.

Cavalcanti et al. [12] described the *minisphenoidal approach*.

#### 2018

Takeda et al. [81] described the *distal transsylvian keyhole approach*.

Kocaman et al. [43] described the *modified lateral supraorbital approach*.

#### 2020

Chandra et al. [14] described the *fronto-orbital variant of the supraorbital keyhole craniotomy (f-SOKHA).*

Martinez-Perez et al. [51] described the *extradural minipterional approach*, which was also separately described by Mura et al. [58] using the same name.

Sattur et al. [76] described the *extended lateral orbital approach***.**

#### 2022

Nerntengian et al. [60] described the *mini-spheno-supraorbital craniotomy.*

Rychen et al. [75] described the *Sylvian keyhole approach*.

Secere et al. [78] described the *modified osteoplastic pterional craniotomy.*

### Difference in skin incision

There were two broad types of incisions that are described. The first involves an incision in the eyebrow or eyelid that may extend beyond the orbit into the temporal fossa. There are 14 articles [1, 6, 10, 14, 20, 25, 48, 56, 57, 64, 72, 74, 76, 82, 83] that describe this type of skin incision. The second type of incision is curvilinear in shape and is either behind the hairline of the temporal fossa and forehead, on the hairline, or slightly anterior. There are 28 articles [2, 7, 8, 12, 15, 17, 22, 31, 32, 35, 36, 39–43, 45, 51, 58–60, 70, 75, 77–79, 81, 85] that describe this type of skin incision. Two articles [3, 38] describe a bicoronal incision, one article [53] describes question mark/hockey stick incision over the temporal fossa and forehead and another article [55] describes a W-shaped incision.

### Difference in temporalis dissection

There were two common types of temporalis dissection. The most common method involved incising through all layers of the temporalis fascia and muscle and splitting the temporalis muscle with subperiosteal dissection, described in 18 articles [1, 6, 14, 15, 20, 32, 35, 43, 55–57, 64, 72, 74, 76, 79, 82, 83]. The other common method was described in 12 articles [2, 7, 31, 39, 40, 42, 51, 53, 58, 60, 81, 85] and involved an interfascial or subfascial dissection technique with mobilisation of the muscle separately in a different direction. Four articles [12, 17, 22, 36] describe a single myocutaneous flap. Three articles [41, 77, 78] describe an osteoplastic craniotomy. One article [75] describes a T-shaped incision with subperiosteal dissection of each limb, and another article [70] describes incising the muscle along the attachment to the STL and dissecting subperiosteally. Four articles [3, 10, 38, 48] did not describe the method of temporalis dissection.

### Difference in anatomical location of craniotomy

**Fig. 2:**
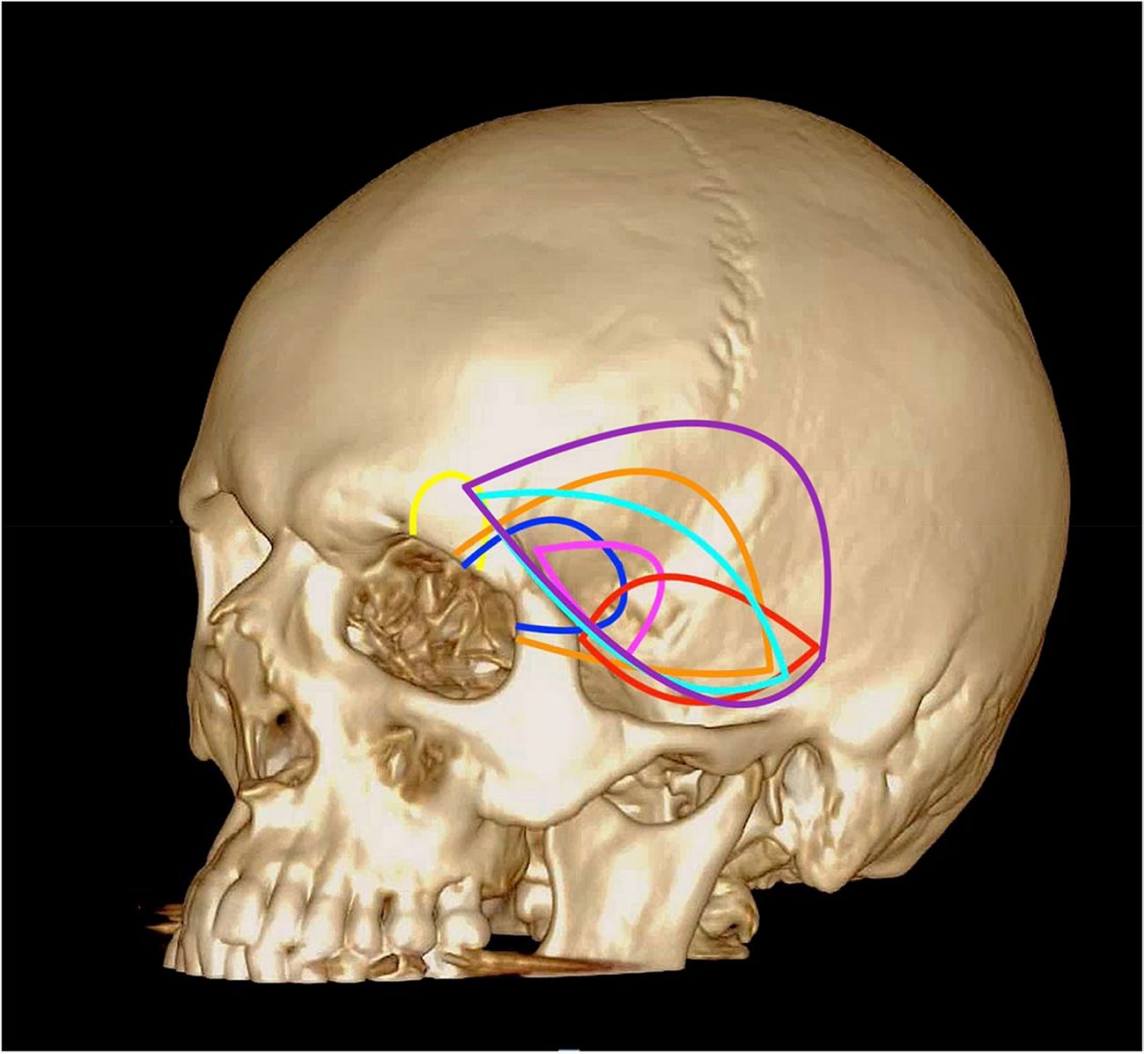
Schematic illustration demonstrating the anatomical locations of the described craniotomies. Each coloured line represents a different anatomical location for the reviewed craniotomies: Cyan, fronto-spheno-temporal; dark blue, fronto-spheno-zygomatic; pink, fronto-sphenoidal; yellow, frontal; orange, fronto-spheno-temporo-zygomatic; red, temporo-sphenoidal; purple, fronto-spheno-parieto-temporal

A total of seven locations for a craniotomy are described (Fig. 2). Nineteen articles [2, 12, 15, 22, 32, 35, 39, 42, 51, 53, 56, 59, 70, 76–78, 81, 83, 85] described a craniotomy that involved removing bone from the fronto-spheno-temporal area (cyan). Eight articles [1, 6, 20, 25, 31, 38, 48, 79] described a craniotomy that involved removing bone from the fronto-spheno-zygomatic area (dark blue). Nine articles [8, 14, 36, 45, 55, 57, 60, 82, 83] described a craniotomy that involved removing bone from the fronto-sphenoidal area (pink). Five articles [10, 43, 64, 72, 74] described a craniotomy involving only frontal bone (yellow). Three articles [3, 7, 40] described a fronto-spheno-temporo-zygomatic craniotomy (orange). One article [17] described a temporo-sphenoidal craniotomy (red), an article [41] described a fronto-spheno-parieto-temporal craniotomy (purple). One article [75] described a fronto-temporal bone removal, which was not able to be demonstrated on Fig. 2.

### Difference in approach

The majority of articles described a combination of subfrontal and/or transsylvian approaches. Twenty articles [1, 6–8, 10, 14, 20, 38, 42, 43, 45, 48, 57, 64, 70, 72, 74, 79, 82, 83] described a pure subfrontal approach. Thirteen articles [2, 12, 15, 17, 35, 51, 53, 55, 56, 58, 59, 75, 81] describe a purely transsylvian approach. Twelve articles [22, 25, 31, 32, 36, 39–41, 60, 76, 78, 85] described both subfrontal and transsylvian approaches. One article [3] described a subfrontal, transsylvian and subtemporal approach.

### Study quality

Twenty-six of the included articles were deemed of good methodological quality. Sixteen articles were deemed of moderate quality and five articles were deemed poor quality (refer to Table 4 for the full assessment).

**Table 4:** Quality assessment consensus table

Paper	Literature review	Surgical technique	Case series	Evaluation	Overall quality
Abdel Aziz et al. 2011	Good	Good	Good	Good	Good
Ahn et al. 2017	Poor	Moderate	Good	Good	Moderate
Al-Mefty 1987	Good	Good	Poor	Good	Moderate
Andaluz et al. 2003	Moderate	Good	Poor	Good	Moderate
Andaluz et al. 2008	Good	Good	Good	Good	Good
Andrade-Barazarte et al. 2017	Good	Good	Good	Good	Good
Brydon et al. 2008	Good	Moderate	Good	Good	Good
Cavalcanti et al. 2017	Good	Good	Good	Good	Good
Chandra et al. 2020	Good	Poor	Good	Good	Moderate
Chehrazi et al. 1992	Good	Good	Good	Good	Good
Cheng et al. 2006	Good	Good	Good	Good	Good
Dare 2001	Good	Moderate	Good	Good	Good
Deshaies et al. 2015	Good	Moderate	Good	Good	Good
Dzhindzhikhadze et al. 2016	Good	Moderate	Good	Good	Good
Figueiredo et al. 2007	Good	Moderate	Poor	Good	Moderate
Figueiredo et al. 2006	Good	Good	Poor	Good	Moderate
Harland et al. 1996	Good	Moderate	Good	Good	Good
Hernesniemi et al. 2005	Good	Good	Poor	Good	Moderate
Jane et al. 1982	Good	Good	Poor	Good	Moderate
Kang et al. 2003	Moderate	Poor	Poor	Poor	Poor
Kelleher et al. 2005	Good	Good	Good	Good	Good
Kim et al. 2011	Good	Moderate	Poor	Good	Moderate
Kim et al. 2016	Moderate	Poor	Poor	Poor	Poor
Kocaman et al. 2018	Good	Good	Good	Good	Good
Lazukova et al. 2016	Good	Poor	Poor	Good	Poor
Mandel et al. 2017	Good	Moderate	Good	Good	Good
Martinez-Perez et al. 2020	Good	Good	Good	Good	Good
Mocco et al. 2013	Good	Moderate	Good	Good	Good
Mori et al. 2007	Good	Good	Good	Good	Good
Mori et al. 2008	Good	Moderate	Poor	Good	Moderate
Mori et al. 2011	Good	Good	Good	Good	Good
Mura et al. 2020	Good	Moderate	Good	Good	Good
Nathal et al. 2005	Good	Good	Poor	Good	Moderate
Nerntengian et al. 2022	Good	Moderate	Good	Good	Good
Paladino et al. 1998	Good	Good	Good	Good	Good
Petridis et al. 2017	Poor	Poor	Poor	Good	Poor
Ramos-Zuniga et al. 2002	Good	Good	Good	Good	Good
Reisch et al. 2003	Good	Good	Poor	Poor	Poor
Rychen et al. 2022	Good	Good	Poor	Good	Moderate
Sattur et al. 2020	Good	Good	Good	Good	Good
Schlitt et al. 1989	Good	Moderate	Poor	Good	Moderate
Secer et al. 2022	Good	Moderate	Good	Good	Moderate
Steiger et al. 2001	Good	Good	Good	Good	Good
Takeda et al. 2018	Good	Good	Poor	Good	Moderate
Tang et al. 2013	Good	Good	Good	Good	Good
Van Lindert et al. 1998	Good	Good	Good	Good	Good
Yasargil et al. 1976	Good	Good	Poor	Good	Moderate

## Discussion

### A proposition for standardised nomenclature

We have described the exhaustive number of techniques, variations and modifications described for fronto-spheno-temporal or pterional craniotomy. Ultimately, these 47 different articles can be distilled into two types of incisions (frontotemporal or periorbital), two types of craniotomies (frontal or a craniotomy involving sphenoid such as fronto-spheno-temporal/fronto-spheno-zygomatic/fronto-sphenoidal) and two approaches (subfrontal or transsylvian). There are several modifiers that have been described at each of these stages such as the length of the incision, management of the underlying muscle, size of the craniotomy, an osteotomy including the supraorbital bar [1, 6, 7, 20, 32, 40, 72], osteoplastic craniotomy [41, 77, 78] or use of an extradural anterior clinoidectomy [8, 51, 58].

Minipterional, pterional keyhole, sphenoid ridge approach, Sylvian keyhole, distal transsylvian approach, thumbsize pterional, minisphenoidal and minimal invasive pterional keyhole are just a few of the many names that were identified in the systematic review. More recently, extended minipterional and nanopterional have been described, but did not meet inclusion criteria of this review [47, 50].

These articles create the impression that aneurysm surgery is an outside-in concept. It implies that surgeons must learn a large number of these craniotomies and their named modifications to treat certain target lesions. This concept is misleading as it is really considered from inside-out, despite not being articulated in this way. The majority of the included articles focus on naming the type of craniotomy, or minor variation in the exposure. They all still largely target the same anatomical zone.

In clinical practice, surgeons examine the lesion to be treated and then consider the target zone they want exposed. This will vary by surgical goal (multiple aneurysms, complexity, requirement for deconstructive or reconstructive techniques) and surgeon experience (increasing experience gives confidence to manage with a smaller exposure). The surgeon then considers what approaches grant them the necessary degrees of freedom and visibility, before finally considering the craniotomy and skin incision that will enable that approach. By having so many articles, it is easy to overlook this essential component of designing the neurosurgical procedure for your patient. When the first description appeared of the minipterional in 2007, Bernard George highlighted the importance of considering the general principle of the location of the pterional craniotomy to the Sylvian fissure, rather than to ‘precisely describe a surgical technique and to consider any little change or variation as a new technique’.

The inside-out concept is more evident in articles describing different techniques in skull base oncology surgery. The focus of these articles is to describe the anatomical exposure at the target zone, the degree of freedom offered by the approach and the surgeon’s unique way of exposing these structures. The surgeon then offers case examples of pathology that can be addressed safely using the technique described. It is far less common in the skull base oncology literature to find articles describing all of the different neoplasms addressed through an extremely specific craniotomy or named modification. Moreover, the authors will present an article that is based on their experience treating a specific pathology and the variety of approaches they have had to utilise to achieve their surgical goals. Shifting towards this paradigm for vascular neurosurgery would be beneficial.

We advocate for more simplified and consistent nomenclature. Craniotomies were traditionally described based on the calvarial bones incorporated into the craniotomy. Atlay et al. describe this in detail during a historical perspective on the frontotemporosphenoidal (FTS) approach [5]. Variations were named when major changes occurred compared to the FTS approach, such as Hakuba et al. [33] orbitozygomatic infratemporal approach, Drake [13] half and half approach, and Pellerin et al. [69] orbitofrontomalar approach.

We propose a standardised and intuitive nomenclature to simplify the way these approaches are described, similar to the original description based on the overlying calvarial bones (Fig. 2).

Based on the ‘inside-out’ concept, we advocate to describe all approaches based on the target zone, subdural or subarachnoid corridor being used to approach the aneurysm, the constituent bones included in the craniotomy and the location of the associated incision. *A major approach-related consideration is the target zone to be exposed*, which is defined by the specific aneurysm and surgical goal. Using this new system of nomenclature applied to an ACoA aneurysm first requires understanding of the anatomic and therapeutic considerations specific to the aneurysm. What direction does the dome of the aneurysm project? If superiorly projecting, do you consider interhemispheric, subfrontal or transsylvian? If inferiorly projecting, do you consider subfrontal or transsylvian? Which side is the dominant A1 and will there be benefit from approaching the aneurysm on that side? Once the target zone and approach corridor are defined the constituent bones involved in the craniotomy become evident and the associated incision to grant this access can be planned based on surgeon experience. If additional areas of bone removal are required, such as an anterior clinoidectomy, these modifiers are defined along with the bones being removed in the craniotomy. This is because the removal of these structures is being done to access the target zone, and therefore is entirely determined by the target zone and corridor required to treat the aneurysm. The additional removal of bone and the shape and type of incision have considerable variation.

The inside-out concept can be considered as a pyramid with the target zone at the apex of the pyramid, as it is essentially the aneurysm morphology to be treated. The corridor, craniotomy and skin incision are then listed in order of increasing degrees of freedom.

**Fig. 3:**
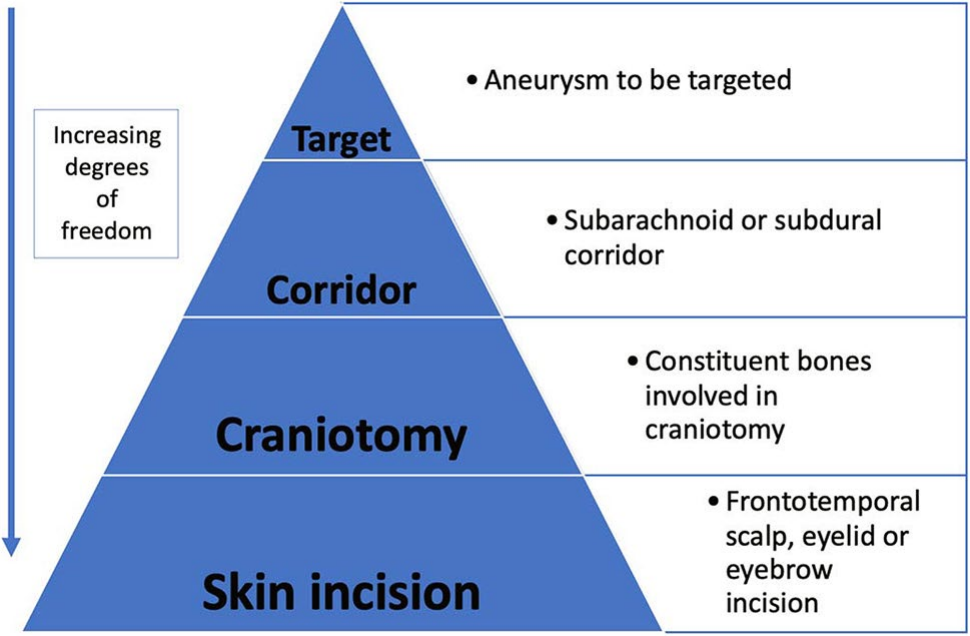
Proposal of how to apply the ‘inside-out’ concept for aneurysm surgery and a way to simplify the approach related nomenclature

For example, an inferiorly projecting ACoA aneurysm would be defined by the following: the target zone (inferiorly projecting ACoA aneurysm), corridor (right/left-sided subfrontal), craniotomy (frontal/fronto-sphenoidal/fronto-spheno-temporal), incision (eyebrow/frontotemporal scalp) (Fig. 3). The long hand description could then be an inferiorly projecting ACoA aneurysm approached subfrontally through an eyebrow incision and right-sided fronto-sphenoidal craniotomy. A short hand description would be to simply describe the bones involved; a right-sided fronto-sphenoidal approach.

Another example describing a right M1/2 aneurysm would be defined by the following: the target zone (right M1/2 aneurysm with a dome projecting frontally and a long M1), corridor (right transsylvian), craniotomy (fronto-sphenoidal/fronto-spheno-temporal/spheno-temporal), incision (frontotemporal). Therefore, the long hand description would be as follows: a right M1/2 aneurysm approached transsylvian through a frontotemporal scalp incision and fronto-spheno-temporal craniotomy. Associated short hand description being a right-sided fronto-spheno-temporal approach.

Ultimately the success of an operation is determined by the surgeon tailoring their incision, craniotomy and approach to the patient and their pathology. The cut-off at which size truly affects patient-outcome is currently unknown, particularly because surgeon experience will likely have a greater affect then the size of the incision or the amount of bone removed. The use of a standardised nomenclature would help clarify this.

It is interesting to note that the International Society on Minimally Invasive Neurosurgery recently published a consensus statement regarding standardising nomenclature in an effort to define the variety of keyhole procedures that are described in the literature [44]. The result of this standardisation will allow for more effective comparisons to be examined.

## Limitations

A potential limitation of this article is the narrow scope that is applied to only consider articles relating to vascular neurosurgery. By excluding all the articles focusing on oncological neurosurgery, a large number of important articles will have been excluded. However, the purpose of this review was to highlight the trend in the vascular neurosurgery literature of renaming minor and inconsequential modifications. This trend is not as prevalent in oncological neurosurgery, which is why the review did not consider these articles.

## Conclusion

We have demonstrated a systematic review of modifications and variations for the pterional craniotomy that exists in the literature. There is an exhaustive number of minor variations that do not serve to expand the general principles of aneurysm surgery. We advocate for a simplified and standardised nomenclature when considering surgical management of aneurysms that can describe the incision and craniotomy type, but should focus on the approach and target zone exposure.

## Data Availability

Nothing to disclose.

## Abbreviations

ACoA: Anterior communicating artery
MCA: Middle cerebral artery
DACA: Distal anterior cerebral artery
STA: Superficial temporal artery
ICA: Internal carotid artery
LSO: Lateral supraorbital
PComA: Posterior communicating artery

## Code availability

Nothing to disclose.
